# Association Between Antioxidant Nutrients, Oxidative Stress-Related Gene Polymorphism and Skeletal Fluorosis in Guizhou, China

**DOI:** 10.3389/fpubh.2022.849173

**Published:** 2022-05-13

**Authors:** Na Tao, Lianhong Li, Qing Chen, Zhongming Sun, Qinglin Yang, Dafang Cao, Xun Zhao, Fangfang Zeng, Jun Liu

**Affiliations:** ^1^Department of Pharmacy, Affiliated Hospital of Zunyi Medical University, Zunyi, China; ^2^Department of Preventive Medicine, School of Public Health, Zunyi Medical University, Zunyi, China; ^3^Department of Chronic Diseases, Center for Diseases Control and Prevention of Zhijin County, Zhijin, China; ^4^Department of Epidemiology, School of Medicine, Jinan University, Guangzhou, China

**Keywords:** skeletal fluorosis, antioxidant nutrients, oxidative stress, gene polymorphism, cross-sectional study

## Abstract

**Background:**

Oxidative stress plays an important role in the pathogenesis of endemic fluorosis. We analyzed associations between oxidative stress-related gene polymorphisms (PON1 rs662, CAT rs769217, rs2300182, and SOD2 rs11968525) and skeletal fluorosis, and examined potential gene–environment interactions with dietary vitamin C, vitamin E, zinc, and selenium intake.

**Methods:**

A cross-sectional study was conducted in the Zhijin County, Guizhou Province of China. Skeletal fluorosis was identified according to the Chinese Diagnostic Criteria of Endemic Skeletal Fluorosis. Dietary information was assessed through face-to-face interviews by trained interviewers using a 75-item food frequency questionnaire. The genotype was detected by high throughput TaqMan-MGB RT-PCR technology. Odds ratios (ORs) and 95% CIs were calculated using an unconditional logistic regression model.

**Results:**

Intake of vitamin E, zinc, and selenium was found to be inversely associated with the risk of skeletal fluorosis. The multivariable-adjusted ORs were 0.438 (95% CI: 0.268 to 0.715, *P-*trend < 0.001) for vitamin E, 0.490 (95% CI: 0.298 to 0.805, *P-*trend = 0.001) for zinc, and 0.532 (95% CI: 0.324 to 0.873, *P-*trend = 0.010) for selenium when comparing the highest with the lowest quartile. The relationship for vitamin C was not observed after adjustment for risk factors. Furthermore, participants with PON1 rs662 AA genotype had a significantly decreased risk of skeletal fluorosis compared with those with the GG genotype (OR = 0.438, 95% CI: 0.231 to 0.830). GG + AG genotype carriers were 2.212 times more likely to have skeletal fluorosis than AA carriers (OR = 2.212, 95% CI: 1.197 to 4.090). Compared with AA carriers, AG carriers had a 2.182 times higher risk of skeletal fluorosis (OR = 2.182, 95% CI: 1.143 to 4.163). Although we observed the risk of skeletal fluorosis was higher with a lower intake of antioxidant nutrients, the potential interactions between nutrient intake and genetic polymorphisms were not observed.

**Conclusion:**

Participants with a higher intake of vitamin E, zinc, and selenium have a lower likelihood of skeletal fluorosis. In addition, the PON1 rs662 polymorphism is related to skeletal fluorosis.

## Introduction

Fluorosis has become a public health problem worldwide. Excessive fluoride intake can disrupt the processes of bone formation and resorption, which may lead to bone turnover disorders and further result in skeletal fluorosis (1). More than 16.1 million dental fluorosis patients and 1.8 million patients with skeletal fluorosis have been reported in the Chinese province of Guizhou. Patients with skeletal fluorosis experience persistent pain and bone and joint damage. They are physically limited and cannot perform labor-intensive work and may even become permanently disabled. Skeletal fluorosis decreases the quality of life and the patients become a social and economic burden on society (2, 3). Therefore, it is imperative to prevent and control skeletal fluorosis.

Oxidative stress has been reported to participate in the pathogenesis of endemic fluorosis (4, 5). Oxidative stress was defined as a disturbance in the balance between the production of reactive oxygen species (ROSs) and antioxidant defenses, including enzymatic and non-enzymatic systems. Non-enzymatic systems are composed of dietary vitamin C, vitamin E, zinc, and selenium (6). Some studies have shown that antioxidant nutrients, such as vitamin C and vitamin E, had a protective effect against fluorosis (7–9), while other studies have not found this effect (10). Conflicting results may be due to states of oxidative stress being elevated in part of the participants. It was thought that susceptibility to fluorosis was related to gene polymorphism (11–13). However, these studies did not consider modification by genetics.

Antioxidant enzymes, such as glutathione peroxidase (GPx), catalase (CAT), superoxide dismutase (SOD), and paraoxonase (PON), are an important barrier to oxidative damage (4, 14, 15). It was reported that single nucleotide polymorphisms (SNPs) of genes encoding the antioxidant enzymes contribute to genetic changes that affect the activity and function of the enzymes (16, 17). PON is a family (PON-1, PON-2, PON-3) of antioxidant enzymes with anti-inflammatory functions. PON-1 RR Q192R (rs662) is a common functional SNP in PON1. The PON-1 rs662 polymorphism was associated with higher enzymatic activity, which may lead to different disease susceptibility (18). Studies reported that CAT and SOD enzyme activities decreased in a fluorosis patient group compared with a control group (19, 20). Studies reported evidence that coal-burning fluorosis was related to decreased SOD activity and gene expression (17, 21). The above studies indicate that oxidative stress is not only affected by exogenous antioxidants, but that gene polymorphism also plays a role. In addition, some studies reported oxidative stress-related gene polymorphisms in PON 1, SOD, and CAT were associated with bone health (14, 22–24). Deng et al. found that the SOD2 gene played a significant role in BMD variation and pathogenesis of osteoporosis and observed the strongest association signals at SNP rs11968525 (25). Thus, oxidative stress-related gene polymorphisms might be associated with skeletal fluorosis.

Although dietary antioxidants were important to assist with decreasing oxidative stress, the circle level of antioxidants and oxidative stress were affected by SNP of PON1, SOD, GPX, etc. (15, 26, 27). Some studies have shown that dietary intake of vitamin C, vitamin E, and selenium were interacted with SNP on disease (27–30). A previous study reported PON1 polymorphisms to modify the association between lycopene and oxidative stress parameters and bone turnover markers, and thus moderated the risk of osteoporosis (22). Therefore, oxidative stress-related gene polymorphisms may modify the association between antioxidant nutrients and skeletal fluorosis. Therefore, we investigated associations between the SNPs PON1 rs662, CAT rs769217, rs2300182, and SOD2 rs11968525 and skeletal fluorosis. We further examined potential gene interactions with dietary vitamin C, vitamin E, zinc, and selenium intake.

## Subjects and Methods

### Study Subjects

A population-based cross-sectional study was conducted between July and August 2015 in a coal-burning area of Zhijin County in the Guizhou Province, China. A two-stage, clustered random sampling method was used in this study. The three towns of Chadian, Chengguan, and Puweng were randomly selected from 10 towns in Zhijin County. Then, we randomly further selected 4 villages from each selected town. The 12 villages selected for the study were Dazai, Ganhe, Gaofeng, Guihua, Guohua, Hehua, Hualuo, Jiangyan, Moda, Shangzai, Yutang, and Xianfeng. Participants who have lived in Zhijin County for at least 10 years and aged 18–75 years were recruited through village doctors and the Center for Disease Control and Prevention (CDC) from the randomly selected villages (31). Participants were excluded if they had a prior history of cancer, coronary heart disease, stroke, gout, or kidney disease. They were also excluded if their dietary habits had manifestly changed during the previous 5 years, or if they had chronic diseases that might affect their dietary habits, such as gastritis, diabetes, and hypertension. In addition, the participants with incomplete questionnaire information were also excluded. A total of 894 participants were successfully interviewed and 165 subjects with no blood samples were excluded from the study. Finally, only 729 participants were included in this analysis. In addition to the questionnaire, each participant also received a clinical examination. Skeletal fluorosis was identified according to the Chinese Diagnostic Criteria of Endemic Skeletal Fluorosis (WS192-2008, China) (32).

This study was conducted in accordance with the guidelines of the Declaration of Helsinki and was proved by the Medical Ethics Committee of Zunyi Medical University (No. 2014-1-003). Written informed consent was obtained from all the participants.

### Data Collection Pertaining to Diet and Lifestyle

Interviews were conducted by trained interviewers who administered a structured questionnaire in a personal interview. The content of the questionnaire included: (1) socio-demographic characteristics (age, gender, ethnicity, marital status, and education level); (2) lifestyle habits (smoking, drinking alcohol, drinking tea, sedentary frequency, vitamin supplement consumption, use of improved stoves, domestic fuel type, use of coal to roast grains and chilies, and washing dry grains and chilies before use); (3) dietary habits in the year before the interview; (4) relevant disease history (hypertension, diabetes, gout, kidney disease, cancer, heart-related diseases, and stroke). Smokers were defined as individuals who smoked at least five packs of cigarettes a year. Alcohol drinkers were defined as participants who drank at least once a week for at least 6 months. Those who drank tea at least twice weekly were defined as tea drinkers. Bodyweight and height were measured with the participant in light clothing, without shoes by previously trained field researchers. The BMI (kg/m^2^) was calculated using weight and height measures.

A 75-item food frequency questionnaire (FFQ) was used to investigate dietary intake in the year before the subjects by trained interviewers during a face-to-face interview. The FFQ included commonly consumed food groups (cereal products, vegetables, fruits, red and processed meat, poultry, fish, shrimp, egg, dairy products, legumes, fungus, algae, nuts, beverages, and soups), and the intake frequency (never, per year, per month, per week, or per day), and the weight of each ingested food. Energy and nutrient intakes were calculated using the Chinese Food Composition Database (33). The validity and reproducibility of the FFQ have been assessed (34).

### Real-Time Polymerase Chain Reaction Genotyping

Venous blood samples of 5 ml were collected in an EDTA anticoagulant tube from each participant on the day of investigation, and stored at −70°C until use. Genomic DNA was extracted using a whole blood DNA extraction kit (Tiangen Biotech, Beijing, China) according to the manufacturer's instructions. Genotyping was performed with high throughput TaqMan-MGB RT-PCR technology. The genotyping was performed on a Roche Lightcycler® 480 platform Software Real-time Fluorescence Quantitative PCR Instrument (Roche, Applied Biosystems). The PCR reaction system was a volume of 6 μl: 0.25 μl TaqMan universal PCR mixture, 0.25 μl of SNP genotyping mixture, 4.5 μl of dd H_2_O, and 1 μl of DNA. PCR amplification conditions were as follows: initial heating at 95°C for 4 min, followed by 40 cycles of 95°C for 7 s and 60°C for 40 s. Two blank controls were set for every 96 well plates. We repeated genotyping at random for 10% of the sample.

### Statistical Analysis

The data were coded and doubly entered by two data clerks into Epi-Data version 3.1 to avoid clerical errors using side-by-side comparison, and the data were then exported to SPSS for windows version 18 statistical software. The chi-squared (χ^2^) test was used to detect whether the genotype distribution satisfied the Hardy–Weinberg equilibrium among the two groups.

Dietary intake of vitamin C, vitamin E, zinc, and selenium showed skewed distribution, which was classified into quartiles based on the total distribution among the two groups, and the lowest quartile was used as the reference category. The association between the risk of skeletal fluorosis and nutrients or genetic polymorphism was examined using odds ratios (ORs) and 95% CI, which were calculated using the unconditional logistic regression model. The potential interactions between nutrient intake and genetic polymorphism in a dominant genetic model were examined in the multivariate unconditional logistic regression model by adding interactive terms. Age, gender, ethnicity, marital status, education level, smoking, alcohol drinking, tea-drinking, improved stove use, fuel type and using coal to roast grains and chilies, washing dry grains and chilies before use, total energy intake, BMI, sedentary frequency, and vitamin supplement consumption were all added to the model. All the tests of significance were two-sided. Statistical significance was determined at the *p* < 0.05 level.

## Results

### General Characteristics

**Figure 1:**
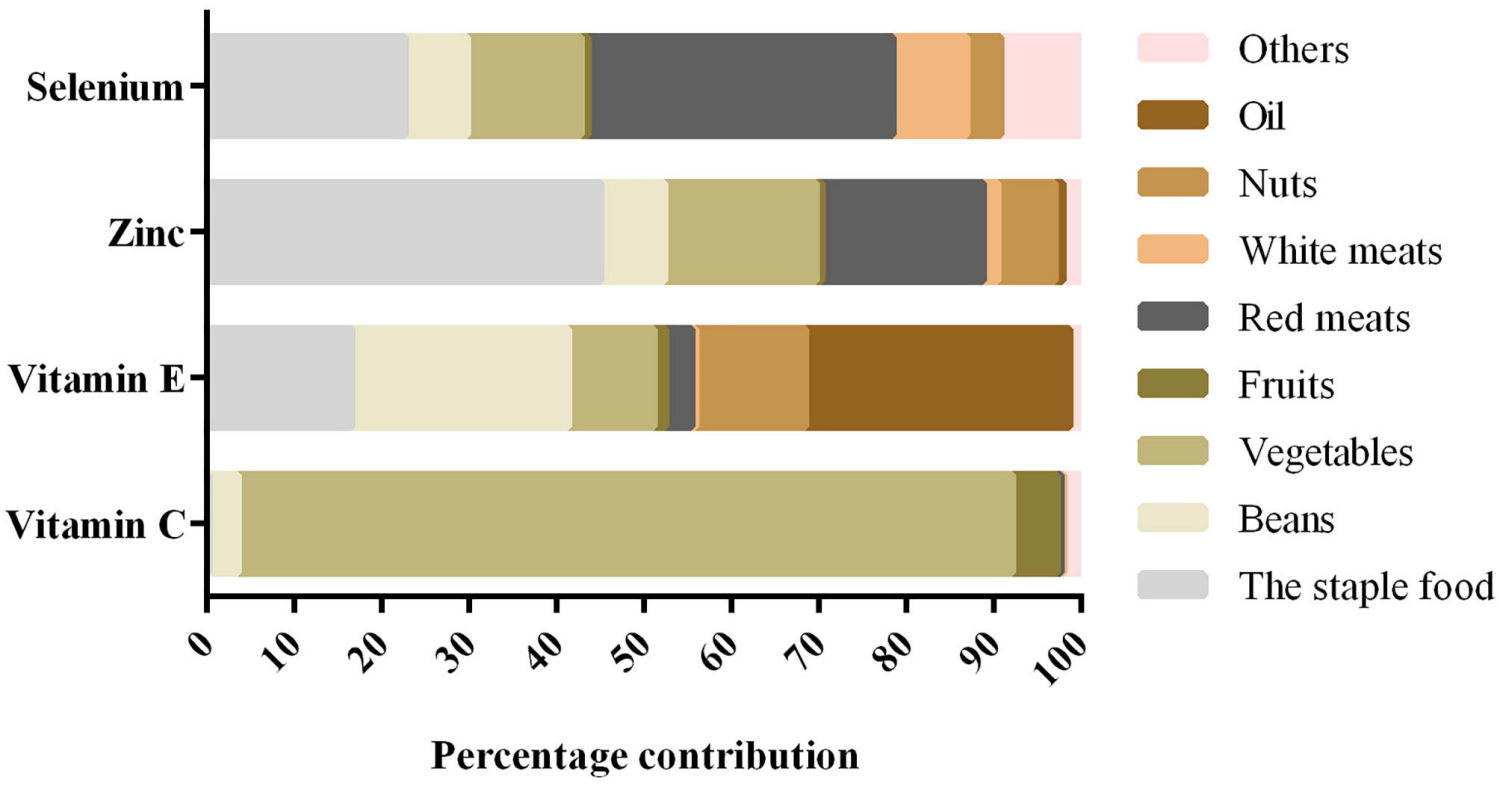
Foods contributing to intake for antioxidant nutrients in the participants.

Of the 729 subjects who participated in this study, 37.9% were diagnosed with skeletal fluorosis. Among the 276 patients with skeletal fluorosis included 136 women (49.28%) and 140 men (50.72%), with a mean age of 55.05 ± 9.98 years. Among the 453 patients without skeletal fluorosis included 250 women (55.19%) and 203 men (44.81%), with a mean age of 46.32 ± 13.02 years. The socio-demographic characteristics of the participants are presented in Table 1. The main food sources of antioxidant nutrients are shown in Figure 1. Vegetables and oil were the main sources of vitamin C and vitamin E, respectively. Zinc and selenium were predominantly consumed from the staple food and red meats, respectively.

**Table 1:** General characteristics of study populations.

**Characteristics**	**Non-skeletal fluorosis (*n* = 453)**	**Skeletal fluorosis (*n* = 276)**	**Total**
Age, year (Mean ± SD)	46.32 ± 13.02	55.05 ± 9.98	49.62 ± 12.68
Gender, *n* (%)			
Female	250 (55.19)	136 (49.28)	386 (52.95)
Male	203 (44.81)	140 (50.72)	343 (47.05)
Smoker, *n* (%)	183 (40.40)	119 (43.12)	302 (41.43)
Alcohol drinker, *n* (%)	134 (29.58)	84 (30.43)	218 (29.90)
Tea drinker, *n* (%)	174 (38.41)	101 (36.59)	275 (37.72)
Sedentary frequency, *n* (%)	6 (1.32)	8 (2.90)	14 (1.92)
Vitamin supplement, *n* (%)	5 (1.10)	2 (0.72)	7 (0.96)
Improved stove, *n* (%)	345 (76.16)	204 (73.91)	549 (75.31)
Using coal to roast grains and chilis, *n* (%)	248 (54.75)	159 (57.61)	407 (55.83)
Washing dry grains andchilies before use, *n* (%)	431 (95.14)	254 (92.03)	685 (93.96)
Fuel type, *n* (%)			
Raw coal	285 (62.91)	153 (55.43)	438 (60.08)
Mixed coal	87 (19.21)	72 (26.09)	159 (21.81)
Firewood	16 (3.53)	10 (3.62)	26 (3.57)
Others	65 (14.35)	41 (14.86)	106 (14.54)
Ethnicity, *n* (%)			
Han	236 (52.10)	165 (59.78)	401(55.01)
Miao	47 (10.38)	32 (11.59)	79 (10.84)
Buyi	73 (16.11)	18 (6.52)	91(12.48)
Others	97 (21.41)	61 (22.10)	158 (21.67)
Marital status, *n* (%)			
Married	381 (84.11)	230 (83.33)	611 (83.81)
Unmarried	31 (6.84)	8 (2.90)	39 (5.35)
Widowed	41 (9.05)	38 (13.77)	79 (10.84)
Education level, year *n* (%)			
≤ 6	183 (40.40)	169 (61.23)	352 (48.29)
6–9	167 (36.87)	87 (31.52)	254 (34.84)
9–12	78 (17.22)	19 (6.88)	97 (13.31)
≥12	25 (5.52)	1 (0.36)	26 (3.57)
	Median (25th, 75th)	Median (25th, 75th)	
Total energy intake (kcal/d)	2552.13 (1914.06, 3307.74)	2613.02 (1997.52, 3529.87)	2583.64 (1969.93, 3402.92)
BMI (kg/m^2^)	22.82 (20.17, 25.76)	22.35 (20.27, 24.94)	22.56 (20.18, 25.62)
Vitamin C (mg/d)	142.85 (97.58, 207.65)	130.23 (83.51, 188.76)	138.67 (90.15, 201.61)
Vitamin E (mg/d)	41.96 (30.03, 57.82)	32.45 (24.61, 47.27)	37.72 (27.55, 53.68)
Zinc (mg/d)	20.10 (15.46, 24.64)	17.37 (13.72, 22.19)	18.78 (14.71, 23.39)
Selenium (ug/d)	38.66 (27.57, 54.61)	32.63 (23.69, 47.19)	36.41 (25.57, 52.27)

### Association Between Dietary Nutrient Intake and Skeletal Fluorosis

As shown in Table 2, intake of vitamin E, zinc, and selenium was inversely associated with the risk, of skeletal fluorosis. The multivariable-adjusted ORs were 0.438 (95% CI: 0.268 to 0.715, *P-*trend < 0.001) for vitamin E, 0.490 (95% CI: 0.298 to 0.805, *P-*trend = 0.001) for zinc, and 0.532 (95% CI: 0.324 to 0.873, *P-*trend = 0.010) for selenium when comparing the highest with the lowest quartile. Intake of vitamin C was not associated with the risk of skeletal fluorosis after adjusting for potential confounding factors.

**Table 2:** Association between dietary nutrients intake and skeletal fluorosis.

**Quartile of nutrients**	**Median**	**Non-SF**	**SF**	**OR (95% CI)^a^**	**OR (95% CI)^b^**
**Vitamin C (mg/d)**					
Q1	60.76	100	82	1.00	1.00
Q2	116.93	116	66	0.694 (0.456–1.056)	0.806 (0.503–1.291)
Q3	166.07	114	69	0.738 (0.486–1.121)	0.847 (0.532–1.348)
Q4	277.55	123	59	0.585 (0.382–0.896)^*^	0.641 (0.398–1.031)
*P*-trend				0.024	0.094
**Vitamin E (mg/d)**					
Q1	22.33	87	95	1.00	1.00
Q2	32.10	108	74	0.627 (0.414–0.950)^*^	0.702 (0.444–1.109)
Q3	44.31	122	61	0.458 (0.300–0.699)^***^	0.531 (0.331–0.849)^**^
Q4	67.85	136	46	0.310 (0.199–0.482)^***^	0.438 (0.268–0.715)^**^
*P*-trend				<0.001	<0.001
**Zinc (mg/d)**					
Q1	12.02	93	89	1.00	1.00
Q2	16.98	104	78	0.784 (0.518–1.185)	0.911 (0.575–1.445)
Q3	21.22	123	60	0.510 (0.334–0.779)^**^	0.546 (0.338–0.882)^*^
Q4	28.04	133	49	0.385 (0.248–0.597)^***^	0.490 (0.298–0.805)^**^
*P*–trend				<0.001	0.001
**Selenium (ug/d)**					
Q1	20.50	94	88	1.00	1.00
Q2	31.50	111	71	0.683 (0.451–1.036)	0.770 (0.481–1.231)
Q3	43.41	118	65	0.588 (0.387–0.895)^*^	0.652 (0.404–1.050)
Q4	65.71	130	52	0.427 (0.277–0.659)^***^	0.532 (0.324–0.873)^*^
*P*-trend				<0.001	0.010

*SF, skeletal fluorosis*.

a *Crude OR (95% CI) without further adjustment*.

b *Adjust for age, gender, ethnicity, marital status, education level, smoking, alcohol drinking, tea–drinking, improved stove use, fuel type and using coal to roast grains and chilies, washing dry grains and chilis before use, total energy intake, BMI, sedentary frequency, and vitamin supplement consumption*.

* *P < 0.05*,

** *P < 0.01*,

*** *P < 0.001*.

### Distribution Characteristics of Genotypes

Testing for deviation from Hardy–Weinberg equilibrium was performed using the χ^2^ test. The genotype distributions of PON1 rs662, CAT rs769217, rs2300182, and SOD2 rs11968525 polymorphisms of both patient groups were found to be in Hardy–Weinberg equilibrium (*P*>0.05) (Supplementary Table 1). The PON1 rs662 polymorphism was related to the risk of skeletal fluorosis. After adjusting for confounding factors, the relationship remained the same. Participants with the AA genotype had a significantly decreased risk of skeletal fluorosis than those with the GG genotype (OR = 0.438, 95% CI: 0.231 to 0.830). Moreover, CAT rs769217, rs2300182, and SOD2 rs11968525 polymorphisms were not associated with skeletal fluorosis (Table 3).

**Table 3:** Frequency distributions of genotypes in the non-skeletal fluorosis and skeletal fluorosis.

**Genotype**	**Non-skeletal fluorosis, *n* (%)**	**Skeletal fluorosis, *n* (%)**	**OR (95% CI)^a^**	**OR (95%CI)^b^**
PON1 rs662				
GG	200 (44.15)	128 (46.38)	1.00	1.00
AG	197 (43.49)	130 (47.10)	1.031 (0.754–1.411)	0.940 (0.667–1.324)
AA	56 (12.36)	18 (6.52)	0.502 (0.282–0.893)^*^	0.438 (0.231–0.830)^*^
CAT rs769217				
CC	130 (28.70)	77 (27.90)	1.00	1.00
CT	217 (47.90)	132 (47.83)	1.027 (0.720–1.465)	0.920 (0.623–1.359)
TT	106 (23.40)	67 (24.28)	1.067 (0.704–1.618)	0.948 (0.602–1.494)
CAT rs2300182				
AA	323 (71.30)	191 (69.20)	1.00	1.00
AT	120 (26.49)	74 (26.81)	1.043 (0.742–1.466)	0.951 (0.655–1.382)
TT	10 (2.21)	11 (3.99)	1.860 (0.776–4.462)	1.931 (0.740–5.037)
SOD2 rs11968525				
GG	298 (65.78)	171 (61.96)	1.00	1.00
AG	126 (27.81)	87 (31.52)	1.203 (0.864–1.677)	1.292 (0.896–1.864)
AA	29 (6.40)	18 (6.52)	1.082 (0.583–2.006)	1.027 (0.527–2.002)

a *Crude OR (95% CI) without further adjustment*.

b *Adjust for age, gender, ethnicity, marital status, education level, smoking, alcohol drinking, tea–drinking, improved stove use, fuel type and using coal to roast grains and chilies, washing dry grains, and chilis before use*.

* *P < 0.05*.

### Association Between Different Genetic Models of Oxidative Stress-Related Genes and Skeletal Fluorosis

The PON1 rs662, CAT rs2300182, rs769217, and SOD2 rs11968525 SNPs were divided into four different genetic models listed in Table 4. Two univariate models (dominant and accumulative) showed that the SNP PON1 rs662 was associated with the risk of skeletal fluorosis, after adjusting for confounding factors. GG+AG genotype carriers were 2.212 times more likely to have skeletal fluorosis than those with AA (OR = 2.212, 95% CI: 1.197 to 4.090). Compared with AA genotype carriers, AG genotype carriers had 2.182 times higher risk of skeletal fluorosis (OR = 2.182, 95% CI: 1.143 to 4.163). However, the four different genetic models did not show a relationship to skeletal fluorosis for SNPs CAT rs2300182, rs769217, and SOD2 rs11968525.

**Table 4:** Distribution and risk estimation of SNP polymorphisms in non-skeletal fluorosis and skeletal fluorosis.

**Gene locus**	**Inheritance model**	**Non–skeletal Fluorosis, *n* (%)**	**Skeletal fluorosis, *n* (%)**	**OR (95% CI)^a^**	**OR (95% CI)^b^**
PON1 rs662	superdominance				
	AG	197 (43.49)	130 (47.10)	1.00	1.00
	GG + AA	256 (56.51)	146 (52.90)	0.864 (0.640–1.167)	0.932 (0.671–1.295)
	dominant				
	AA	56 (12.36)	18 (6.52)	1.00	1.00
	GG + AG	397 (87.64)	258 (93.48)	2.022 (1.162–3.517)^*^	2.212 (1.197–4.090)^*^
	recessive				
	GG	200 (44.15)	128 (46.38)	1.00	1.00
	AG + AA	253 (55.85)	148 (53.62)	0.914 (0.677–1.235)	0.833 (0.599–1.158)
	accumulation				
	AA	56 (22.13)	18 (12.16)	1.00	1.00
	AG	197 (77.87)	130 (87.84)	2.053 (1.155–3.650)^*^	2.182 (1.143–4.163)^*^
CAT rs769217	superdominance				
	CT	217 (47.90)	132 (47.83)	1.00	1.00
	CC + TT	236 (52.10)	144 (52.17)	0.997 (0.739–1.345)	1.061 (0.764–1.474)
	dominant				
	TT	106 (23.40)	67 (24.28)	1.00	1.00
	CC + CT	347 (76.60)	209 (75.72)	1.049 (0.739–1.490)	1.000 (0.682–1.467)
	recessive				
	CC	130 (28.70)	77 (27.90)	1.00	1.00
	CT + TT	323 (71.30)	199 (72.10)	0.961 (0.689–1.341)	0.929 (0.645–1.339)
	accumulation				
	TT	106 (32.82)	67 (33.67)	1.00	1.00
	CT	217 (67.18)	132 (66.33)	0.962 (0.662–1.400)	0.990 (0.653–1.500)
CAT rs2300182	superdominance				
	AT	120 (26.49)	74 (26.81)	1.00	1.00
	TT + AA	333 (73.51)	202 (73.19)	0.984 (0.701–1.380)	1.079 (0.744–1.564)
	dominant				
	TT	10 (2.21)	11 (3.99)	1.00	1.00
	AA + AT	443 (97.79)	265 (96.01)	1.839 (0.771–4.388)	0.511 (0.197–1.327)
	recessive				
	AA	323 (71.30)	191 (69.20)	1.00	1.00
	AT + TT	130 (28.70)	85 (30.80)	1.106 (0.798–1.533)	1.024 (0.716–1.464)
	accumulation				
	TT	10 (7.69)	11 (12.94)	1.00	1.00
	AT	120 (92.31)	74 (87.06)	0.561 (0.227–1.384)	0.503 (0.153–1.648)
SOD2 rs11968525	superdominance				
	AG	126 (27.81)	87 (31.52)	1.00	1.00
	GG + AA	327 (72.19)	189 (68.48)	0.837 (0.604–1.160)	0.776 (0.541–1.113)
	dominant				
	AA	29 (6.40)	18 (6.52)	1.00	1.00
	GG + AG	424 (93.60)	258 (93.48)	0.980 (0.534–1.801)	1.056 (0.548–2.037)
	recessive				
	GG	298 (65.78)	171 (61.96)	1.00	1.00
	AG + AA	155 (34.22)	105 (38.04)	1.181 (0.865–1.611)	1.238 (0.879–1.743)
	accumulation				
	AA	29 (18.71)	18 (17.14)	1.00	1.00
	AG	126 (81.29)	87 (82.86)	1.112 (0.582–2.128)	1.325 (0.646–2.716)

a *Crude OR (95% CI) without further adjustment*.

b *Adjust for age, gender, ethnicity, marital status, education level, smoking, alcohol drinking, tea drinking, improved stove use, fuel type and using coal to roast grains and chilies, washing dry grains, and chilis before use*.

* *P < 0.05*.

### Interaction Between Oxidative Stress-Related Gene Polymorphisms and Nutrient Intake

As presented in Table 5, patients with diets below the median nutrient intake level with the SNP PON1 rs662 and GG + AG genotype had a higher risk of skeletal fluorosis, compared with AA genotype carriers. The multivariable-adjusted ORs were 4.076 (95% CI: 1.362 to 12.198) for vitamin C, 2.925 (95% CI: 1.163 to 7.356) for vitamin E, 3.392 (95% CI: 1.197 to 9.613) for zinc, and 3.720 (95% CI: 1.420 to 9.743) for selenium. However, the interaction between nutrients and the PON1 polymorphism has not been associated with the risk of skeletal fluorosis. Moreover, there was no significant interaction effect between nutrient intake and the CAT rs2300182 and SOD2 rs11968525 polymorphisms, related to the risk of skeletal fluorosis (Supplementary Tables 2–4).

**Table 5:** Interaction between PON1 rs662 polymorphism and vitamin C, vitamin E, zinc, and selenium.

**Variables**	**Non-skeletal fluorosis, *n* (%)**	**Skeletal fluorosis, *n* (%)**	**OR (95%CI)^a^**	**OR (95%CI)^b^**	***P* for interaction**
**Vitamin C**					0.179
< median					
AA	25 (11.57)	6 (4.05)	1.00	1.00	
GG + AG	191 (88.43)	142 (95.95)	3.098 (1.238–7.751)^*^	4.076 (1.362–12.198)^*^	
≥median					
AA	31 (13.08)	12 (9.38)	1.00	1.00	
GG + AG	206 (86.92)	116 (90.63)	1.455 (0.719–2.942)	1.661 (0.739–3.735)	
**Vitamin E**					0.399
< median					
AA	22 (11.28)	10 (5.88)	1.00	1.00	
GG + AG	173 (88.72)	160 (94.12)	2.035 (0.935–4.429)	2.925 (1.163–7.356)^*^	
≥median					
AA	34 (13.18)	8 (7.55)	1.00	1.00	
GG + AG	224 (86.82)	98 (92.45)	1.859 (0.831–4.163)	1.710 (0.695–4.208)	
**Zinc**					0.251
< median					
AA	20 (10.15)	7 (4.17)	1.00	1.00	
GG + AG	177 (89.85)	161 (95.83)	2.599 (1.071–6.308)^*^	3.392 (1.197–9.613)^*^	
≥median					
AA	36 (14.06)	11 (10.19)	1.00	1.00	
GG + AG	220 (85.94)	97 (89.81)	1.443 (0.705–2.953)	1.593 (0.684–3.709)	
**Selenium**					
< median					0.217
AA	24 (11.65)	8 (5.03)	1.00	1.00	
GG + AG	182 (88.35)	151 (94.97)	2.489 (1.087–5.701)^*^	3.720 (1.420–9.743)^**^	
≥median					
AA	32 (12.96)	10 (8.55)	1.00	1.00	
GG + AG	215 (87.04)	107 (91.45)	1.593 (0.755–3.361)	1.334 (0.568–3.132)	

a *Crude OR (95% CI) without further adjustment*.

b *Adjust for age, gender, ethnicity, marital status, education level, smoking, alcohol drinking, tea drinking, improved stove use, fuel type and using coal to roast grains and chilies, washing dry grains and chilis before use, total energy intake, BMI, sedentary frequency, and vitamin supplement consumption*.

* *P < 0.05*,

** *P < 0.01*.

## Discussion

This study showed that the intake of vitamin E, zinc, and selenium in the patient diet was inversely associated with the occurrence of skeletal fluorosis. It also showed that the PON1 rs662 SNP was related to the occurrence of skeletal fluorosis.

Oxidative stress is the main contributing mechanism to coal-burning fluorosis, and antioxidant nutrients play an important role in coal-burning fluorosis (35). In this study, we found that a higher intake of antioxidant nutrients is associated with lower occurrence of skeletal fluorosis. A previous study found that vitamin E had a positive protective effect on experimental chronic fluorosis in rats (7). Another animal study also reported that vitamin E could significantly reduce the damage to reproductive function in male rabbits exposed to fluoride (36). Liu et al. (37) reported that vitamin C, vitamin E, folic acid, and other antioxidants can enhance the body's antioxidant capacity, and thus, prevent the occurrence of skeletal fluorosis. An epidemiological study showed that a diet rich in antioxidant nutrients could reduce the risk of fluorosis (38). Vitamin E is an important antioxidant in the body and is well-known for its antioxidant activity and free radical scavenging function, which works by scavenging free radicals, active oxygen, and inhibiting lipid peroxidation (39). Thus, vitamin E is believed to exert its protective effect mainly by destroying the oxygen-free radicals produced by fluoride that destroy cells (36). It has also been found to protect hard tissue from fluoride damage by preventing excessive accumulation of fluoride in bones and teeth (40). Vitamin E has been shown to prevent fluorosis-induced spermatogenic cell apoptosis by inhibiting oxidative stress-mediated JNK and ERK signaling pathways (39). The aforementioned studies may partially explain the protective effects of vitamin E against skeletal fluorosis.

We also found that the dietary intake of zinc and selenium was inversely associated with skeletal fluorosis. Zinc is an essential trace element for the human body and a component of certain enzymes and proteins. In agreement with our findings, an Indian study showed that compared with the control group, the serum zinc level in the fluorosis group was significantly lower (41). Moreover, Ersoy et al. (42) conducted a case-control study of 30 patients with fluorosis, and 30 healthy controls showed that chronic fluorosis was related to lowered serum zinc levels. They considered that the significant decrease of serum zinc in the fluorosis patient group may be related to the increased excretion of zinc in urine, the decrease of plasma proteins, such as albumin, and the inability of albumin to transport zinc in its binding form. Similarly, selenium is a typical trace element that is beneficial to human and animal health. An animal experiment showed that a certain concentration of selenium reduced toxicity caused by fluorine (43). Epidemiological studies also showed that, compared with healthy children in low-risk fluorosis areas, the levels of calcium and zinc in the blood of children with chronic fluorosis were 5 times lower and the levels of selenium were 2 times lower (9). Selenium could have an antioxidant function by scavenging free radicals and repairing membrane damage, and it reduced apoptosis induced by fluoride (44, 45). In addition, selenium alleviated NaF-induced apoptosis by increasing the expression of HSP70 and alleviated oxidative stress by regulating the levels of SIRT1 and antioxidant enzymes (46). Miao et al. (47) observed that a certain concentration of selenium could enhance the activity of SOD and GPX antioxidant enzymes, reduce the toxic effect of fluoride, improve liver function, and inhibit apoptosis to a certain extent. These results suggested that fluorosis was not only related to antioxidant nutrients but could also involve other causes.

PON1 is located on chromosome 7q21.3-q22.1 and consists of 354 amino acids. PON1 can hydrolyze a variety of substrates, such as organophosphorus, aromatic esters, lactones, low-density lipoprotein (LDL), and cholesterol, to prevent oxidative damage and lipid peroxidation (48). PON1 is considered to be involved in the development of oxidative stress-related diseases, such as osteoporosis, atherosclerosis, coronary heart disease, diabetes, and metabolic syndrome (49–52). In this study, we first reported that PON1 rs662 polymorphism was associated with the risk of skeletal fluorosis. The carriers of the AA genotype had a lower risk of skeletal fluorosis than carriers of GG, GG + AG, and AG genotypes. Some studies explored the relationship between the PON1 rs662 polymorphism and bone health. In agreement with our findings, a case-control study from China reported that the GG genotype and the G allele of the rs662 polymorphism were closely related to the increased risk of ankylosing spondylitis (53). However, another case-control study found that the carriers of the AA genotype of rs662 in PON1 were more susceptible to osteonecrosis of the femoral head than GG genotype carriers (OR = 2.53, 95% CI: 1.05 to 6.07) (48). In addition, there were studies which had confirmed that the PON1 gene polymorphism was associated with osteoporosis (23, 54). A reasonable explanation for the inconsistency of these studies may be because of the different distribution of PON1 polymorphisms in different diseases, which leads to different disease susceptibility. More and more evidence indicated that there was a biochemical relationship between lipid oxidation and skeletal biology. Increased lipid oxidation causes oxidative stress and reduces Wnt signaling, thereby reducing osteoblast differentiation and survival, and inducing osteoclast differentiation through the cAMP-mediated pathway (55). Mackinnon et al. (22) found that the PON1 gene polymorphism was associated with multiple markers of bone conversion, and PON1 could catalyze the decomposition of peroxides, reduce the accumulation of lipid peroxidation products, and reduce the effect of oxidative stress on bone formation. Although the PON1 polymorphism can affect bone metabolism by participating in lipid oxidation and reducing oxidative stress, the exact mechanism needs to be confirmed by further studies.

In this study, we also analyzed the interactions between nutrient intake and gene polymorphisms. A previous study showed that PON1 polymorphisms modified the association between serum concentrations of lycopene and oxidative stress parameters and bone turnover markers and therefore, decreased the risk of osteoporosis (22). A population-based study showed that high-selenium levels interacted with potential genes associated with oxidative stress (56). Moreover, two studies showed a statistically significant interaction between SOD2 with dietary intake of vitamin E and selenium affecting the risk of cancer (30, 57). Although PON1 rs662 GG + AG carriers had a higher risk of skeletal fluorosis than the AA genotype at low-antioxidant nutrient levels, none of PON1 rs662, CAT rs2300182, and SOD2 rs11968525 interacted with antioxidant nutrients on skeletal fluorosis. No interaction was observed which may be attributed to the small sample.

This study has some limitations. At first, the study was a cross-sectional study design, the causal relationship could not be determined, although we minimized possible reverse causation by excluding participants with essential changes in dietary habits over the past 5 years. Second, the oxidative stress induced by fluoride was affected by the activity of antioxidant enzymes, and polymorphisms of antioxidant enzyme genes affected enzyme activities. Although we collected blood for genotype identification, we did not measure the activity of antioxidant enzymes in the blood. Third, skeletal fluorosis has a close relationship with lifestyle (37). We only collected sedentary frequency without detailed physical activity level, so only sedentary frequency was adjusted to control for the effect of activity level. Finally, for interaction analysis, the sample size of this study was relatively small and antioxidant nutrients were stratified by median rather than quartiles, which may have obscured statistical differences. Larger sample studies are needed to demonstrate the interactions.

## Conclusion

In summary, this study showed that the participants with a higher intake of vitamin E, zinc, and selenium had a lower likelihood of skeletal fluorosis. In addition, the PON1 rs662 polymorphism is related to the occurrence of skeletal fluorosis. Larger studies are needed to determine whether there is an interaction between PON1 rs662 polymorphism and antioxidant nutrients.

## Data Availability Statement

The datasets used and analyzed during the current study are available from the corresponding author on reasonable request.

## Ethics Statement

The studies involving human participants were reviewed and approved by Medical Ethics Committee of Zunyi Medical University. Written informed consent from the participants' legal guardian/next of kin was not required to participate in this study in accordance with the national legislation and the institutional requirements.

## Author Contributions

JL and FZ designed the study and conducted the study. NT was responsible for data management, analysis, and revised critically for important intellectual content, and completed supplement analysis of data. LL was responsible for writing the first draft of the manuscript and revised it. JL, FZ, and QC critically revised the manuscript. XZ, QY, DC, and ZS carried out the study. All authors participated in data interpretation, review, and approved the final manuscript.

## Funding

This study was supported by the National Natural Science Foundation of China (grant number 81460497 and 82060595) and Guizhou Province Foundation for postgraduate Scientific Research Fund Project YJSKYJJ [2021]191. The funders had no role in study design, data collection and analysis, decision to publish, or preparation of the manuscript.

## Conflict of Interest

The authors declare that the research was conducted in the absence of any commercial or financial relationships that could be construed as a potential conflict of interest.

## Publisher's Note

All claims expressed in this article are solely those of the authors and do not necessarily represent those of their affiliated organizations, or those of the publisher, the editors and the reviewers. Any product that may be evaluated in this article, or claim that may be made by its manufacturer, is not guaranteed or endorsed by the publisher.
