# Supplementary material for: Association Between Antioxidant Nutrients, Oxidative Stress-Related Gene Polymorphism and Skeletal Fluorosis in Guizhou, China
Source: Front Public Health. 2022 May 13;10:849173. doi: 10.3389/fpubh.2022.849173 (PMC9140744; doi:10.3389/fpubh.2022.849173)
Supplement: Supplementary file 1 [file Data_Sheet_1.pdf]

Supplementary Table 1 | The Hardy-Weinberg equilibrium in the non-skeletal fluorosis and skeletal fluorosis

**Genotype**	**Non-skeletal fluorosis, n = 453 (%)**	**Skeletal fluorosis, n = 276 (%)**
rs662		
GG	200 (44.15)	128 (46.38)
AG	197 (43.49)	130 (47.10)
AA	56 (12.36)	18 (6.52)
$\chi 2$	0.195	2.123
P-$_{HWE}$	0.907	0.346
rs769217		
CC	130 (28.70)	77 (27.90)
CT	217 (47.90)	132 (47.83)
TT	106 (23.40)	67 (24.28)
$\chi 2$	0.358	0.262
P-$_{\mathrm{HWE}}$	0.836	0.877
rs2300182		
AA	323 (71.30)	191 (69.20)
AT	120 (26.49)	74 (26.81)
TT	10 (2.21)	11 (3.99)
$\chi 2$	0.052	0.659
P-$_{\mathrm{HWE}}$	0.974	0.719
rs11968525		
GG	298 (65.78)	171 (61.96)
AG	126 (27.81)	87 (31.52)
AA	29 (6.40)	18 (6.52)
$\chi 2$	3.868	0.988
P-$_{\mathrm{HWE}}$	0.145	0.610

Supplementary Table 2 | Interaction between CAT rs769217 polymorphism and Vitamin C, Vitamin E, Zinc, Selenium

**Variables**	**Non-skeletal fluorosis, n (%)**	**Skeletal fluorosis, n (%)**	**OR (95%CI) [a]**	**OR (95%CI) [b]**	***P* for interaction**
CAT rs769217					
Vitamin C					0.751
<median					
TT	53 (24.54)	36 (24.32)	1.00	1.00	
CC++CT	163 (75.46)	112 (75.68)	1.012 (0.622-1.646)	1.169 (0.667-2.048)	
≥median					
TT	53 ((22.36)	31 (24.22)	1.00	1.00	
CC++CT	184 (77.64)	97 (75.78)	0.901 (0.543-1.496)	0.920 (0.514-1.649)	
Vitamin E					0.695
<median					
TT	39 (20.00)	41 (24.12)	1.00	1.00	
CC++CT	156 (80.00)	129 (75.88)	0.787 (0.479-1.292)	0.961 (0.560-1.649)	
≥median					
TT	67 (25.97)	26 (24.53)	1.00	1.00	
CC++CT	191 (74.03)	80 (75.47)	1.079 (0.640-1.820)	1.101 (0.609-1.988)	
Zinc					0.044
<median					
TT	40 (20.30)	45 (26.79)	1.00	1.00	
CC++CT	157 (79.70)	123 (73.21)	0.696 (0.428-1.133)	0.722(0.420-1.241)	
≥median					
TT	66 (25.78)	22 (20.37)	1.00	1.00	
CC++CT	190 (74.22)	86 (79.63)	1.358 (0.787-2.343)	1.735 (0.930-3.238)	
Selenium					0.156
<median					
TT	43 (20.87)	41 (25.79)	1.00	1.00	
CC++CT	163 (79.13)	118 (74.21)	0.759 (0.466-1.238)	0.783 (0.449-1.364)	
≥median					
TT	63 (25.51)	26 (22.22)	1.00	1.00	
CC++CT	184 (74.49)	91 (77.78)	1.198 (0.711-2.018)	1.387 (0.775-2.484)	

a: Crude OR (95% CI) without further adjustment.

b: Adjust for age, gender, ethnicity, marital status, education level, smoking, alcohol drinking, tea drinking, improved stove use, fuel type and using coal to roast grains and chilis, washing dry grains and chilis before use，total energy intake, BMI, sedentary frequency, vitamin supplement consumption.

Supplementary Table 3 | Interaction between CAT rs2300182 polymorphism and Vitamin C, Vitamin E, Zinc, Selenium

Variables	Non-skeletal fluorosis, n (%)	Skeletal fluorosis, n (%)	OR (95%CI) [a]	OR (95%CI) [b]	*P* for interaction
CAT rs2300182					
Vitamin C					0.564
<median					
TT	5 (2.31)	6 (4.05)	1.00	1.00	
AA++AT	211 (97.69)	142 (95.95)	0.561 (0.168-1.873)	0.808 (0.218-3.000)	
≥median					
TT	5 (2.11)	5 (3.91)	1.00	1.00	
AA++AT	232 (97.89)	123 (96.09)	0.530 (0.151-1.867)	0.379 (0.084-1.700)	
Vitamin E					0.952
<median					
TT	3 (1.54)	5 (2.94)	1.00	1.00	
AA++AT	192 (98.46)	165 (97.06)	0.516 (0.121-2.190)	0.578 (0.129-2.580)	
≥median					
TT	7 (2.71)	6 (5.66)	1.00	1.00	
AA++AT	251 (97.29)	100 (94.34)	0.465 (0.152-1.417)	0.466 (0.125-1.739)	
Zinc					0.539
<median					
TT	4 (2.03)	5 (2.98)	1.00	1.00	
AA++AT	193 (97.97)	163 (97.02)	0.676 (0.178-2.558)	0.886 (0.185-4.239)	
≥median					
TT	6 (2.34)	6 (5.56)	1.00	1.00	
AA++AT	250 (97.66)	102 (94.44)	0.408 (0.129-1.295)	0.422 (0.121-1.477)	
Selenium					0.510
<median					
TT	5 (2.43)	6 (3.77)	1.00	1.00	
AA++AT	201 (97.57)	153 (96.23)	0.634 (0.190-2.117)	0.794 (0.209-3.010)	
≥median					
TT	5 (2.02)	5 (4.27)	1.00	1.00	
AA++AT	242 (97.98)	112 (95.73)	0.463 (0.131-1.631)	0.399 (0.094-1.705)	

a: Crude OR (95% CI) without further adjustment.

b: Adjust for age, gender, ethnicity, marital status, education level, smoking, alcohol drinking, tea drinking, improved stove use, fuel type and using coal to roast grains and chilis, washing dry grains and chilis before use，total energy intake, BMI, sedentary frequency, vitamin supplement consumption.

Supplementary Table 4 | Interaction between SOD2 rs11968525 polymorphism and Vitamin C, Vitamin E, Zinc, Selenium

**Variables**	**Non-skeletal fluorosis, n (%)**	**Skeletal fluorosis, n (%)**	**OR (95%CI) [a]**	**OR (95%CI) [b]**	***P* for interaction**
Vitamin C					0.223
<median					
AA	10 (4.63)	10 (6.76)	1.00	1.00	
GG++AG	206 (95.37)	138 (93.24)	0.670 (0.272-1.652)	0.516 (0.180-1.484)	
≥median					
AA	19 (8.02)	8 (6.25)	1.00	1.00	
GG++AG	218 (91.98)	120 (93.75)	1.307 (0.556-3.076)	1.281 (0.498-3.294)	
Vitamin E					0.428
<median					
AA	14 (7.18)	14 (8.24)	1.00	1.00	
GG++AG	181 (92.82)	156 (91.76)	0.862 (0.399-1.864)	0.865 (0.364-2.057)	
≥median					
AA	15 (5.81)	4 (3.77)	1.00	1.00	
GG++AG	243 (94.19)	102 (96.23)	1.574 (0.510-4.858)	1.513 (0.447-5.122)	
Zinc					0.180
<median					
AA	8 (4.06)	11 (6.55)	1.00	1.00	
GG++AG	189 (95.94)	157 (93.45)	0.604 (0.237-1.539)	0.467 (0.163-1.338)	
≥median					
AA	21 (8.20)	7 (6.48)	1.00	1.00	
GG++AG	235 (91.80)	101 (93.52)	1.289 (0.531-3.129)	1.431 (0.532-3.845)	
Selenium					0.069
<median					
AA	8 (3.88)	11 (6.92)	1.00	1.00	
GG++AG	198 (96.12)	148 (93.08)	0.544 (0.213-1.385)	0.448 (0.157-1.280)	
≥median					
AA	21 (8.50)	7 (5.98)	1.00	1.00	
GG++AG	226 (91.50)	110 (94.02)	1.460 (0.602-3.539)	1.736 (0.658-4.581)	

a: Crude OR (95% CI) without further adjustment.

b: Adjust for age, gender, ethnicity, marital status, education level, smoking, alcohol drinking, tea drinking, improved stove use, fuel type and using coal to roast grains and chilis, washing dry grains and chilis before use，total energy intake, BMI, sedentary frequency, vitamin supplement consumption.
